# Sequencing and Analysis of Three Mycobacterium tuberculosis Genomes of the B0/N-90 Sublineage

**DOI:** 10.1128/MRA.00796-19

**Published:** 2019-09-26

**Authors:** Natalia V. Zakharevich, Marina V. Zaychikova, Kirill V. Shur, Olga B. Bekker, Dmitry A. Maslov, Valery N. Danilenko

**Affiliations:** a Vavilov Institute of General Genetics Russian Academy of Sciences, Moscow, Russia; University of Maryland School of Medicine

## Abstract

We report the draft genome sequences of three Mycobacterium tuberculosis isolates belonging to the B0/N-90 sublineage, EKB34, EKB53, and EKB79. The B0/N-90 sublineage belongs to the prevalent (in Russia) and highly virulent Beijing-B0/W148 sublineage. Isolates EKB34 and EKB79 were obtained from people with immune deficiency.

## ANNOUNCEMENT

Recently, tuberculosis has become more common. The probability of disease manifestation depends on several factors, chief among which are the host immune status and virulence of the pathogen, which are different in different lines (1, 2). Currently, there is a suggestion that the B0/N-90 sublineage (a group belonging to the prevalent [in Russia] and highly virulent Beijing-B0/W148 sublineage of the Beijing family) mainly affects immunocompromised people (3, 4).

In this article, we describe the following isolates from people living in Russia: M. tuberculosis EKB34, isolated from a 49-year-old male, and M. tuberculosis EKB53 and EKB79, isolated from 16- and 27-year-old immunodeficient males, respectively. All isolates are part of the collection described in reference 4 and were genotyped as belonging to the B0/N-90 sublineage by nonsynonymous single-nucleotide polymorphism (SNP) analysis described in reference 3. M. tuberculosis was cultured in Middlebrook 7H9 medium with the addition of oleic acid-albumin-dextrose-catalase (OADC; HiMedia, India) at 37°C for 4 weeks. Genomic DNA was isolated and purified by phenol-chloroform-isoamyl alcohol extraction after enzymatic cell lysis, as described by Belisle et al. (5). The quality of DNA was checked using gel electrophoresis and a Bioanalyzer 2100 system. Genomic DNA libraries were prepared using the NEBNext Ultra II DNA library prep kit for Illumina (New England BioLabs, USA). The raw sequencing data were obtained using a HiSeq 2500 platform (Illumina, USA) in rapid run mode with the HiSeq Rapid SBS kit v2 2 × 100 bp (Illumina). A total of 17,966,286 pairs of reads with an average read length of 111 bases were obtained. Quality check of the reads was done using FastQC v0.11.7 (6). *De novo* genome assembly was performed using SPAdes v3.11 (7), while assembly metrics were calculated with QUAST v5.0.2 (both programs were used with default parameters) (8). The automatic functional annotation results were obtained using the NCBI Prokaryotic Genome Annotation Pipeline. The characteristics of the three sequenced genomes are listed in Table 1.

**TABLE 1:** Characteristics of three M. tuberculosis isolates belonging to the B0/N-90 sublineage

Isolate	WGS (GenBank) accession no.	SRA accession no.	Genome size (bp)	GC content (%)	Coverage (×)	No. of contigs	*N*_50_ value (bp)	No. of contigs >500 bp	Total no. of CDSs^*a*^
EKB34	SRLI00000000	SRR8439237	4,356,659	65.2	46	160	92,498	130	4,269
EKB53	SRLJ00000000	SRR8363334	4,358,808	64.4	169	180	73,246	149	4,268
EKB79	SRLK00000000	SRR8374277	4,357,388	64.6	223	177	72,890	149	4,270

a CDSs, coding sequences.

To identify meaningful differences between isolates of the B0/N-90 sublineage and Beijing-B0/W148 sublineage, the unassembled reads were aligned to the reference genome of M. tuberculosis W-148 (GenBank accession number CP012090) using BWA-MEM, with default settings (9). The pileup was generated by mpileup (-B -f) in SAMtools (10). We used the mpileup2snp (–min-avg-qual 30 –min-var-freq 0.80 –p-value 0.01 –output-vcf 1) command in VarScan 2.3.9 (11) for calling single-nucleotide variants. We found 37 SNPs common to all the B0/N-90 strains and absent in the reference genome, 7 SNPs present in 2 out of 3 strains, as well as a number of unique SNPs present only in one strain (6 for EKB34, 8 for EKB53, and 11 for EKB79). A comparative analysis of virulence-associated genes from the previously developed catalogue (3, 12, 13) showed that besides the previously known lineage-specific mutations (genes *mce3F* [rv1971], A_1229_С [Asp→Ala]; *vapC46* [rv3384c], C_113_G [Ala→Gly]; and *irtB* [rv1349], G_523_A [Ala→Thr]) (3), all three isolates also had a mutation in the Rv3132c gene. This gene is part of the *dosRS* two-component system involved in response to stress conditions; in particular, it regulates the expression of the *hspX* gene, which encodes a protein that plays an important role in regulating the growth of M. tuberculosis under hypoxic conditions (14). However, this mutation is a synonymous one and does not result in an amino acid change. On the contrary, SNPs in the three genes described above may lead to changes in the properties of the encoded proteins, resulting in changes to virulence levels (15, 16).

### Data availability.

The whole-genome shotgun (WGS) assemblies have been deposited in NCBI GenBank. The versions described in this paper are the first versions. The read archives have been deposited in the NCBI SRA. The WGS (GenBank) and SRA accession numbers are listed in Table 1. All of the data are part of BioProject number PRJNA509547.
